# Immunohistochemical basis for FAP as a candidate theranostic target across a broad range of cholangiocarcinoma subtypes

**DOI:** 10.3389/fnume.2024.1480471

**Published:** 2024-11-27

**Authors:** Laura C. Jorgenson, Michael S. Torbenson, Thorvardur R. Halfdanarson, Lionel A. Kankeu Fonkoua, Nguyen H. Tran, Lewis R. Roberts, Rory L. Smoot, Ajit H. Goenka, Scott M. Thompson

**Affiliations:** ^1^ Department of Radiology, Mayo Clinic, Rochester, MN, United States; ^2^ Department of Laboratory Medicine and Pathology, Mayo Clinic, Rochester, MN, United States; ^3^ Division of Medical Oncology, Mayo Clinic, Rochester, MN, United States; ^4^ Division of Gastroenterology and Hepatology, Mayo Clinic, Rochester, MN, United States; ^5^ Division of Hepatobiliary and Pancreas Surgery, Mayo Clinic, Rochester, MN, United States

**Keywords:** cholangiocarcinoma (CCA), fibroblast activation protein (FAP), liver neoplasms, precision medicine, theranostics

## Abstract

**Purpose:**

The aims of this study were to evaluate and compare fibroblast activation protein (FAP) expression and localization in surgically resected cholangiocarcinoma (CCA), primary and metastatic hepatocellular carcinoma (HCC), hepatocellular adenoma (HCA), and focal nodular hyperplasia (FNH), and to identify any association between CCA clinical or pathologic features and FAP expression.

**Materials and methods:**

FAP immunostaining from surgically resected CCA (*N* = 58), primary intrahepatic and extrahepatic metastatic HCC (*N* = 148), HCA (N26), and FNH (*N* = 19) was scored (negative, weak positive, moderate positive or strong positive) from tissue microarrays. FAP expression was compared between groups. CCA FAP expression was compared to clinical and tumor pathology features.

**Results:**

Moderate-strong FAP expression in the tumor stroma was present in 93.1% of CCA, 60.7% of extrahepatic metastatic HCC, 29.6% of primary HCC, 21.1% of FNH, and 11.6% of HCA. Moderate-strong FAP expression in tumor stroma was significantly more prevalent in CCA than HCC (*p* < 0.001), metastatic HCC (*p* = 0.005), HCA (*p* < 0.001) and FNH (*p* < 0.001). FAP was expressed in the stroma of all but one CCA (1.7%), and FAP expression in CCA tumor stroma was not associated with any clinical or tumor pathology features (*p* > 0.05, all).

**Conclusion:**

FAP is expressed in the stroma of a high proportion (93%) of primary CCA independent of patient clinical or tumor pathology features. As such, these data provide the tissue basis for systematically evaluating FAP as a theranostic target across a broad range of CCA subtypes.

## Introduction

Cholangiocarcinomas (CCAs) and gallbladder cancer are known as biliary tract cancers and account for approximately 3% of all gastrointestinal malignancies (1). There are approximately 6,100 cases of intrahepatic and 12,000 cases of extrahepatic cholangiocarcinomas diagnosed annually in the United States (2, 3). There are several risk factors for CCAs, mainly primary sclerosing cholangitis, fibropolycystic liver disease, recurrent pyogenic cholangitis, chronic liver disease, obesity, and metabolic syndrome; however, a specific risk factor is often not identified for many patients. The prognosis for advanced CCA is poor with or without treatment, with the majority of patients having an overall median survival of less than 12 months (4, 5), depending on the disease stage and subtype.

Perihilar tumors comprise about 50% of disease, extrahepatic 40%, and intrahepatic 10% (6). CCAs are adenocarcinomas and often have a dense desmoplastic reaction. Resectability rates are low (7). Importantly, the tumor microenvironment has relative genetic stability and enhances tumor growth, which makes it an important potential target for therapy, given that it cannot develop resistance to therapies through mutation as easily compared to epithelial components (8, 9).

In recent years, fibroblast activation protein (FAP), a cell membrane bound type II serine protease, has emerged as a marker for identifying several tumor types due to its presence in the tumor stroma and has been shown to be associated with worse outcomes in many tumors (10, 11). FAP is overexpressed in tumors with robust desmoplastic reactions and has been validated as a target for molecular diagnostics (12–14). Such desmoplastic reaction is typical of a majority of CCAs. In fact, prior studies using FAP-inhibitor positron emission tomography (PET) radiotracers have shown higher ^68^Ga-FAPI vs. ^18^F-FDG uptake in CCA compared to hepatocellular carcinoma (HCC) (15, 16). This has opened the door for FAP-inhibitor development for tumor-specific targeting small molecule inhibitors and radiopharmaceuticals (17–19). Taken together, FAP may be a candidate theranostic target in CCA with FAP-targeted PET imaging and theranostics.

The aims of this study were to evaluate and compare the protein expression and localization of FAP in surgically resected CCA, primary intrahepatic and extrahepatic metastatic HCC, hepatocellular adenoma (HCA), and focal nodular hyperplasia (FNH), and to identify any association between CCA clinical or pathologic features and FAP expression.

## Materials and methods

This HIPAA-compliant study was conducted following institutional review board (IRB) approval. Tissue Microarrays (TMAs) were constructed from patients who had authorized use of their medical record for research purposes. A waiver of written informed consent was obtained from the IRB.

### Tissue microarrays

Previously constructed TMAs were utilized for the current study and were created using methods previously described (20). TMAs were constructed using the ISEnet Galileo CK4500 instrument (Integrated Systems Engineering srl, Milan, Italy) with 1.0 mm cores from formalin-fixed paraffin-embedded donor blocks stored in the institutional pathology tissue archives selected by a hepatobiliary pathologist (>22 years of experience). Control tissues were included within the array and for orientation including normal prostate, normal liver, ovarian cancer, normal tonsil, and cervical cancer tissues. TMAs from surgically resected CCA (*n* = 58), primary intrahepatic HCC (*n* = 134), extrahepatic metastatic HCC (*n* = 28), HCA (*n* = 26), and FNH (*n* = 19) were used.

### Immunohistochemical staining: anti-fibroblast activation protein

Immunohistochemical (IHC) staining was performed at the Pathology Research Core (Mayo Clinic, Rochester, MN) using the Leica Bond RX stainer (Leica). TMA sections were cut at 5 microns, mounted on charged slides, and dried overnight. For staining, slides were retrieved for 20 min using Epitope Retrieval 1 (Citrate; Leica) and incubated in Protein Block (Dako) for 5 min. The FAP primary antibody (Clone: EPR20021; Abcam ab207178) was diluted to 1:300 in Background Reducing Diluent (Dako) and incubated for 15 min (21, 22).

The detection system used was Polymer Refine Detection System (Leica). This system includes a hydrogen peroxidase block, post primary and polymer reagents, and DAB. Immunostaining visualization was achieved by incubating slides for 10 min in DAB and DAB buffer (1:19 mixture) from the Bond Polymer Refine Detection System. Slides were counterstained for five minutes using a 1:1 mixture of Schmidt hematoxylin (Mayo DLMP Preparation and Processing Laboratory) and molecular biology grade water. Once the immunochemistry process was completed, slides were removed from the stainer and rinsed in tap water for three minutes. Slides were dehydrated in increasing concentrations of ethyl alcohol and cleared in 3 changes of xylene prior to permanent coverslipping in xylene-based medium. Separate slides were stained for hematoxylin and eosin (H&E).

### Pathology review

All H&E and FAP-stained sections were evaluated in a blinded and random fashion by a hepatobiliary pathologist (MST). Histopathology features were analyzed by H&E for tumor grade (well-differentiated, moderately differentiated, poorly differentiated) and histological subtype (small, large, mixed). Both FAP staining intensity and percentage area tumor stroma involved were scored for the FAP-stained sections. The score was a 2-digit number: one for intensity and one for tumor stroma percentage staining. A similar scoring method was previously validated for assessing prostate-specific membrane agent (PSMA) expression by IHC in hepatobiliary tumors (20). The intensity was scored as follows: 0 = none, 1 = weak, 2 = moderate, 3 = strong. The FAP-stained score for percentage staining by area was as follows: 0% staining = 0, 1%–5% staining = 1, 6%–33% staining = 2, 34%–66% staining = 3, and 67%–100% staining = 4. The final interpretation of these two digits was obtained by combining the intensity score and tumor stroma percentage score as follows: 11–12 = negative; 13, 14, 21 = weak positive; 22, 23 = moderate positive; 24, 31–34 = strong positive.

### CCA demographic and clinical data

Demographic and clinical data for the subset of 58 CCA patients were extracted from the electronic medical record.

### Statistical analyses

Study data were collected and managed using REDCap electronic data capture tools (23, 24). Data were analyzed using JMP 16.2.0 (Raleigh, NC). FAP expression by IHC (moderate/strong vs. weak/none and present vs. absent) was compared between CCA, HCC, metastatic HCC, HCA, and FNA groups using Fisher's exact test. Sub-group analysis of the CCA subjects was performed. Categorical variables were compared using the chi-square test or Fisher's exact test. Continuous variables were compared using the one-way ANOVA test or Kruskal-Wallis test. Differences in CCA FAP expression were compared by CCA pathology features (anatomic class, morphology, cellularity type, tumor grade, and size of the involved ducts) using Fisher's exact test. Differences in CCA tumor size were compared by fibroblast activation protein inhibitor (FAPI) expression using a Kruskal-Wallis test. Univariate logistic regression was performed to assess association between FAPI expression with clinical characteristics including absence or presence of choledocholithiasis, cholelithiasis, primary sclerosing cholangitis, cirrhosis, hepatitis B virus, hepatitis C virus, hemochromatosis, inflammatory bowel disease, primary biliary cirrhosis, diabetes, hypertension, alcohol use, cigarette smoking, hyperlipidemia, and asbestos exposure. A *p* < 0.05 was considered statistically significant.

## Results

### FAP protein expression and localization by tumor type

**Figure 1:**
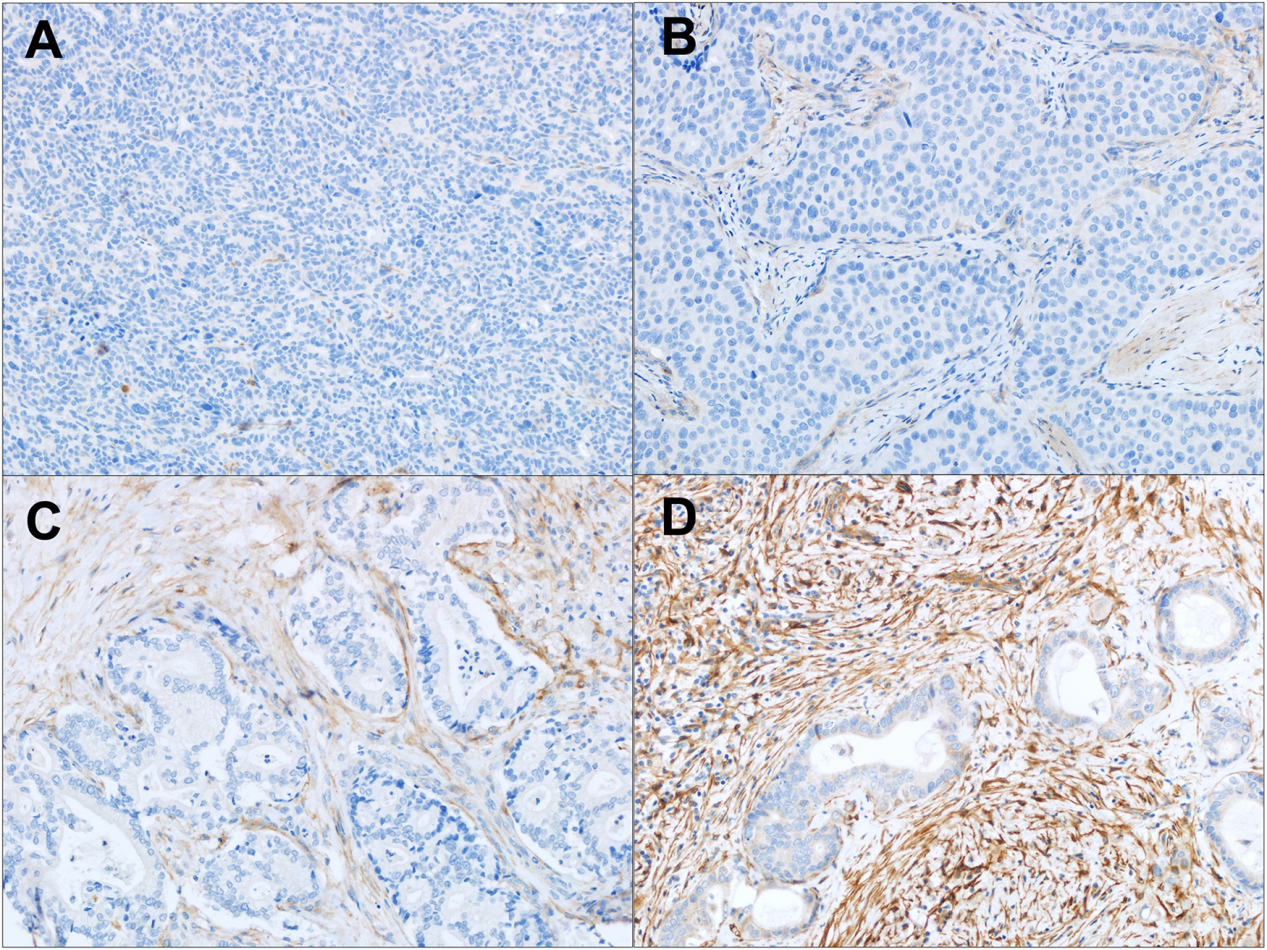
FAP expression in intrahepatic CCA. **(A–D)** Tumor associated stroma FAP staining: **(A)** absent staining in a poorly differentiated CCA; **(B)** weak staining in a poorly differentiated CCA; **(C)** moderate staining in a moderately differentiated CCA; and **(D)** strong staining in a moderately differentiated CCA. Original magnification (20x).

**Figure 2:**
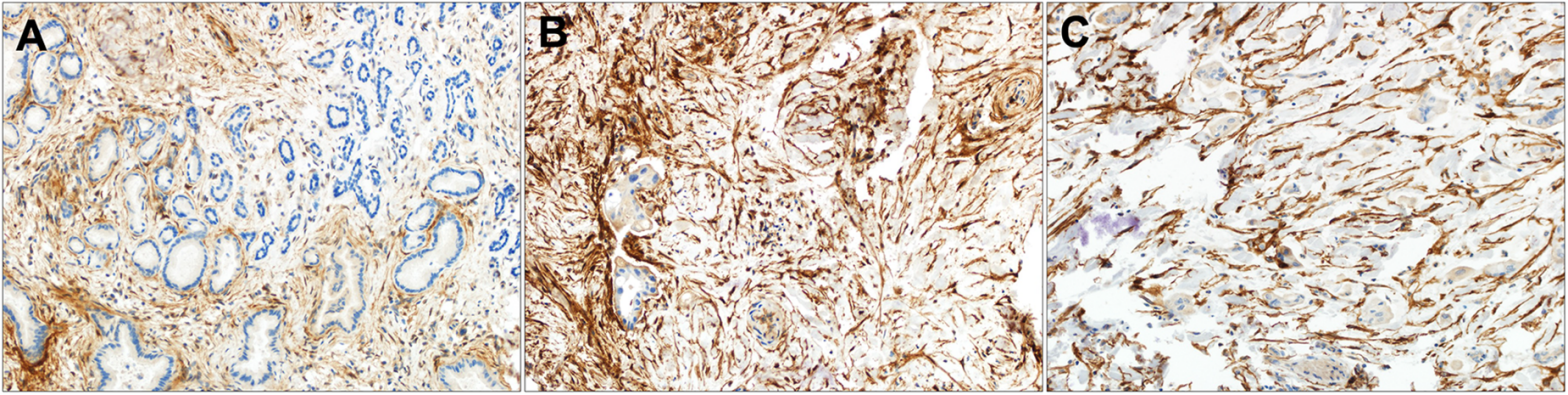
Strong FAP expression in perihilar CCA by tumor grade. **(A–C)** Tumor associated stroma FAP staining in **(A)** well-differentiated, **(B)** moderately differentiated and **(C)** poorly differentiated perihilar CCA. Original magnification (20x).

**Figure 3:**
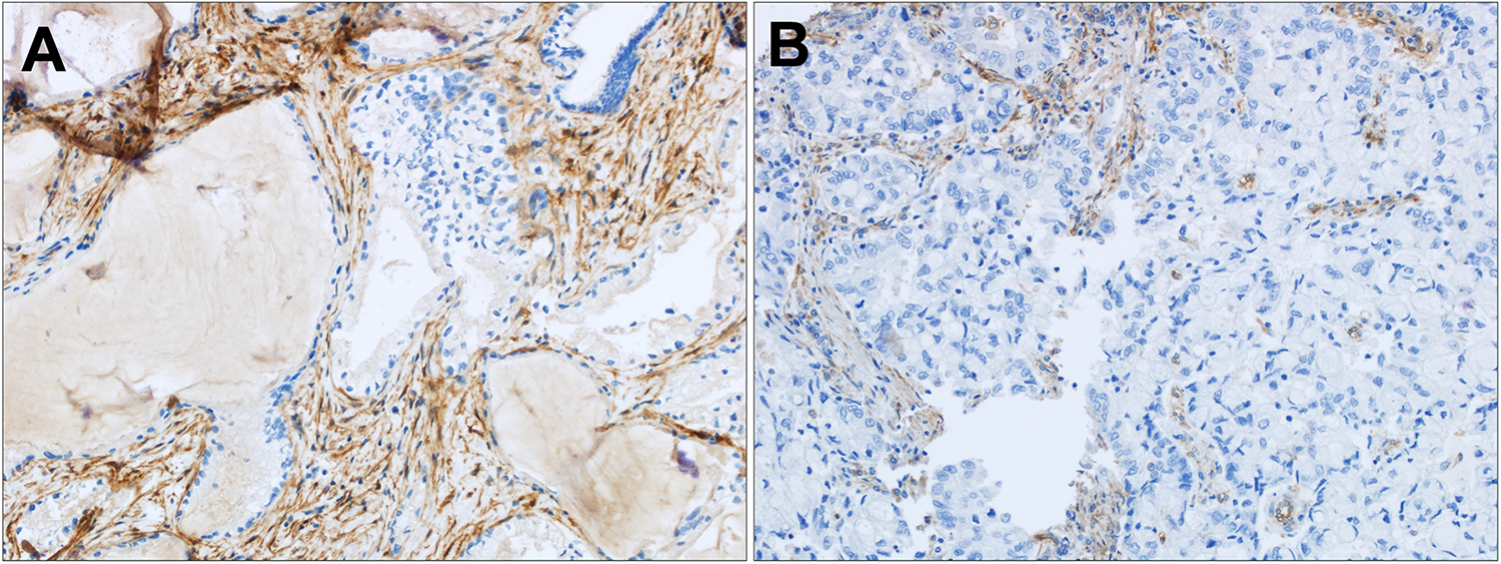
FAP expression in distal and mixed CCA. **(A)** Strong tumor associated stroma FAP staining in a moderately differentiated distal CCA and **(B)** moderate tumor associated stroma FAP staining in a poorly differentiated mixed CCA. Original magnification (20x).

FAP IHC data by tumor type are summarized in Table 1. Moderate-strong FAP expression in the tumor stroma was present in 93.1% (54/58) of CCA (Figures 1–3), 60.7% (17/28) of metastatic HCC (Supplementary Figure S1), 29.6% (42/142) of primary HCC (Supplementary Figure S2), 21.1% (4/19) of FNH (Supplementary Figure S3) and 11.6% (3/26) of HCA (Supplementary Figure S4). FAP expression was not localized to the tumor cells and there was no significant FAP expression in normal background biliary tissue. There was no detectable FAP expression in only one CCA case (Figure 1A, 1.7%), whereas the majority of primary HCC, HCA, and FNH had no detectable FAP expression (57%, 76.9%, and 78.9%, respectively). When comparing moderate/strong vs. negative/weak staining between groups, moderate-strong FAP staining was significantly more prevalent in CCA vs. primary HCC (93.1% v. 29.6%; *p* < 0.0001), metastatic HCC lesions (93.1% v. 60.7%; *p* = 0.0005), FNH (93.1% v. 21.1% *p* < 0.0001), and HCA (93.1% v. 11.6%; *p* < 0.0001), respectively. Moderate-strong FAP staining was significantly more prevalent in metastatic HCC compared to primary HCC (60.7% v. 29.6%; *p* = 0.0023), FNH (60.7% vs. 21.1%; *p* = 0.009) and HCA (60.7% v. 11.6%; *p* = 0.0002), respectively, but there was no difference in FAP staining between primary HCC and FNH or HCA (*p* = 0.59 and *p* = 0.089, respectively). There was no significant difference in FAP expression between FNH and HCA (*p* = 0.43). Similar results were found when comparing present vs. absent FAP staining between groups.

**Table 1:** FAP immunohistochemistry by tumor type.

	Negative	Weak positive	Moderate positive	Strong positive
CCA	1 (1.7%)	3 (5.2%)	6 (10.3%)	48 (82.8%)
HCC	81 (57.0%)	19 (13.4%)	8 (5.6%)	34 (24.0%)
mHCC	9 (32.1%)	2 (7.2%)	8 (28.6%)	9 (32.1%)
HCA	20 (76.9%)	3 (11.5%)	0 (0.0%)	3 (11.6%)
FNH	15 (78.9%)	0 (0.0%)	3 (15.8%)	1 (5.3%)

CCA, cholangiocarcinoma; FNH, focal nodular hyperplasia; HCA, hepatocellular adenoma; HCC, hepatocellular carcinoma; mHCC, metastatic hepatocellular carcinoma.

### FAP by CCA clinical and tumor pathology characteristics

CCA patient clinical and tumor pathology data are summarized in Tables 2 and 3. There was no significant association between FAPI expression and patient age (*p* = 0.33), gender (*p* = 0.76), race (*p* = 0.99), ethnicity (*p* = 0.91), presence of choledocholithiasis (*p* = 0.088), cholelithiasis (*p* = 0.66), primary sclerosing cholangitis (*p* = 0.75), cirrhosis (*p* = 0.49), hepatitis B virus (*p* = 0.85), hepatitis C virus (*p* = 0.053), hereditary hemochromatosis (*p* = 0.94), inflammatory bowel disease (*p* = 0.93), primary biliary cirrhosis (*p* = 0.86), diabetes (*p* = 0.72), obesity (*p* = 0.41), hypertension (*p* = 0.13), alcohol use (*p* = 0.41), cigarette smoking (*p* = 0.52), hyperlipidemia (*p* = 0.58), or asbestos exposure (*p* = 0.83). There was no significant association between FAP expression and CCA tumor size (*p* = 0.20), anatomic location (*p* = 0.15), morphologic subtype (*p* = 0.99), cellularity type (*p* = 0.32), tumor grade (*p* = 0.56), or duct size (*p* = 0.86).

**Table 2:** Demographic and clinical characteristics of 58 patients with surgically resected CCA.

Variable		*N* (%)
Gender	Female	22 (37.9)
	Male	36 (62.1)
Race	White	52 (89.6)
	Black or African American	4 (6.9)
	Asian	1 (1.7)
	American Indian/Alaskan Native	0 (0.0)
	Hispanic or Latino	5 (9.6)
	Unknown/Decline	1 (1.7)
BMI	Mean (standard deviation)	27.3 (5.29)*
Age at Surgery	Mean (standard deviation) in years	61.8 (12.02)*
	Range in years	18.9–84.8**
Clinical	Hypertension	29 (50)
	Alcohol use	23 (39.7)
	Hyperlipidemia	17 (29.3)
	Obesity	16 (28)
	Smoking	11 (19.0)
	Thoratrast exposure	6 (10.3)
	Primary sclerosing cholangitis	3 (5.2)
	Non-alcoholic fatty liver disease	3 (5.2)
	Inflammatory bowel disease	3 (5.2)
	Alpha-1-antitrypsin deficiency	2 (3.6)
	Aflatoxin	2 (3.6)
	Primary biliary cirrhosis	2 (3.4)
	Hepatitis B Virus infection	2 (3.4)
	Hepatitis C Virus infection	2 (3.4)
	Wilson's disease	1 (1.7)
	Liver fluke	1 (1.7)
	Hemochromatosis	1 (1.7)
	Portal hypertension	1 (1.7)
	Asbestos	1 (1.7)

BMI, body mass index.

* Values are mean and (standard deviation).

** Values are a range.

**Table 3:** Tumor characteristics of 58 surgically resected CCAs.

Characteristic		*N* (%)
CCA Anatomic Location	Intrahepatic	37 (63.8)
	Perihilar	19 (32.8)
	Distal	1 (1.7)
	Mixed	1 (1.7)
CCA Morphology	Mass Forming	49 (84.4)
	Periductal	1 (1.7)
	Intraductal	1 (1.7)
	Mixed	6 (10.3)
Tumor Grade	Well Differentiated	3 (5.17)
	Moderately Differentiated	32 (55.2)
	Poorly Differentiated	23 (39.7)
Duct Size	Small	25 (43.1)
	Large	22 (37.9)
	Mixed	8 (13.8)
	Other	3 (5.2)

CCA, cholangiocarcinoma.

## Discussion

The current FAP IHC data confirmed moderate to strong FAP expression in a significantly higher proportion of CCA (93.1%, 54 of 58 patients) compared to other malignant or benign liver tumors. Moreover, FAP was expressed in the tumor stroma of the majority (98.3%, 57 of 58 patients) of CCA, independent of patient clinical or tumor characteristics. Taken together, these data provide the tissue basis for systematically evaluating FAP as a diagnostic and therapeutic target across a broad range of CCA subtypes using FAP-targeted theranostic radiotracers.

These data add to the growing body of evidence supporting current and future clinical trials investigating FAP as both a diagnostic and therapeutic target across multiple tumor types, including biliary tract cancers (16, 25–33). Several studies have shown that CCA cancer-associated fibroblasts induce a robust desmoplastic reaction (34–38). This has in turn supported the development of FAP-targeted PET tracers, which have been used to image at least 28 different cancer types in humans, including CCA (13, 15, 16). When compared to ^18^F-FDG PET, ^68^Ga-FAPI based PET agent performance has been shown to be superior at discrimination of benign vs. malignant lesions, locating primary tumors in metastatic disease (15, 16, 31), and has resulted in restaging in up to 33% of cases (39). Several FAP-targeted PET agents have been used in clinical trials, including ^68^Ga-FAPI-02 (18), ^68^Ga-FAPI-04 (17), ^68^Ga-FAPI-46 (40, 41), and ^68^Ga-FAPI-34 (42). ^18^F-FDG is limited in its ability to stage CCA due to suboptimal liver imaging kinetics (43). Despite high glucose uptake in bile duct epithelial cells, aggressive CCAs are typically not FDG-avid (29, 30, 33, 44, 45). Thus, there is a strong unmet need for improved molecular imaging in CCA.

While a minority of primary HCCs demonstrated moderate to strong FAP expression, a majority of metastatic HCC lesions had moderate to strong FAP expression, suggesting FAP may be a consideration as a theranostic target in metastatic HCC but less so in primary HCC (15, 28, 43). Alternatively, PSMA has been shown to be expressed in a high percentage (>90%) of primary HCCs by IHC, and subsequently confirmed in patients with 68Ga-PSMA PET imaging with greater than 70% of HCC demonstrating grade 3 or 4 PSMA uptake at PET (20). Furthermore, a small percentage of FNH and HCA demonstrated moderate to strong FAP expression, suggesting the FAP-expressing cancer-associated fibroblasts are not entirely specific to malignant lesions. As such, molecular imaging findings will need to be correlated with contrast-enhanced CT or MRI findings for these benign liver tumors.

There are limitations to this study. The IHC was evaluated by a single reader. The correlative data between tissue CCA FAP expression, demographic data, and tumor characteristics are based on TMA and not full tissue sections. This study does not compare FAP expression with CCA genetic mutations or molecular alterations such as clinically relevant, targetable mutations in isocitrate dehydrogenase-1 mutations or fibroblast growth factor receptor-2 fusions or immunotherapy-related indicators such as PD-L1 expression and effector T cell infiltration (46, 47). Additionally, only 1.7% of CCAs were distal in location, and 5% were well-differentiated CCA tumors, thereby limiting the generalizability to these subtypes. Furthermore, only one CCA case was completely negative for FAP expression. This case was a 52-year-old white male with history of cholelithiasis and hepatitis C virus infection, obesity and smoking with a 4.4 cm moderately differentiated, mass forming intrahepatic CCA with small duct size. Given the lack of clinical and tumor features associated with FAP expression in this cohort, the absence of FAP expression in this case cannot be hypothesized. Taken together, while the CCA cases herein are representative of a broad range of CCA subtypes including intrahepatic and perihilar location, moderately and poorly differentiated grades and small and large duct size, further work in larger cohorts of CCA patients will be needed to better understand such outlier cases. Furthermore, due to the retrospective nature, our study does not include any FAP-targeted PET imaging to correlate the degree of FAP expression in the IHC staining data with diagnostic and potentially prognostic significance. Prior studies assessing IHC-PET correlation in HCC with PSMA PET imaging showed that while >90% of HCC were positive for PSMA expression by IHC, >70% of HCC tumors showed high (i.e., grade 3 or 4) uptake at PSMA PET imaging (20). As such, further work is needed to assess FAP expression by IHC and tumor uptake at FAPI PET imaging across a range of benign and malignant liver lesions. Lastly, moderate-strong FAP staining was significantly more prevalent in metastatic HCC compared to primary HCC (60.7% v. 29.6%, respectively). One hypothesis is that there is a greater desmoplastic reaction in the tumor microenvironment with metastatic extrahepatic HCC compared to primary HCC in the liver. Nonetheless, while FAP may be a potential theranostic target in extrahepatic metastatic HCC, further research is needed across both primary intrahepatic and extrahepatic metastatic HCC.

In summary, FAP is expressed in a high proportion (93%) of primary CCA independent of patient clinical or tumor pathology features. As such, these data provide the tissue basis for future studies systematically evaluating FAP as a theranostic target across a broad range of CCA subtypes using paired FAP-targeted PET. FAP-targeted radioligand therapy represents a novel therapeutic approach to targeting the CCA tumor microenvironment in FAP expressing CCA and may be useful in combination with other CCA molecular-targeted or immunotherapies. Such novel diagnostic and therapeutic approaches may help improve outcomes for patients with unresectable CCA.

## Data Availability

The raw data supporting the conclusions of this article will be made available by the authors, without undue reservation.

## Supplementary material

Supplemental Figure S1 FAP expression in metastatic hepatocellular carcinoma (mHCC) (A-C). Negative staining (A), weak positive staining (B), strong positive staining (C). Original magnification (20x).

Supplemental Figure S2 FAP expression in hepatocellular carcinoma (HCC) (A-C). Negative staining (A), weak positive staining (B), strong positive staining (C). Original magnification (20x).

Supplemental Figure S3 FAP expression in fibronodular hyperplasia (A, B). Negative staining (A), moderate positive staining (B). Original magnification (20x).

Supplemental Figure S4 FAP expression in hepatic adenoma (HCA) (A-C). Negative staining (A), weak positive staining (B), strong positive staining (C). Original magnification (20x).
