# Supplementary figures and images for: Immunohistochemical basis for FAP as a candidate theranostic target across a broad range of cholangiocarcinoma subtypes
Source: Front Nucl Med. 2024 Nov 27;4:1480471. doi: 10.3389/fnume.2024.1480471 (PMC11631625; doi:10.3389/fnume.2024.1480471)

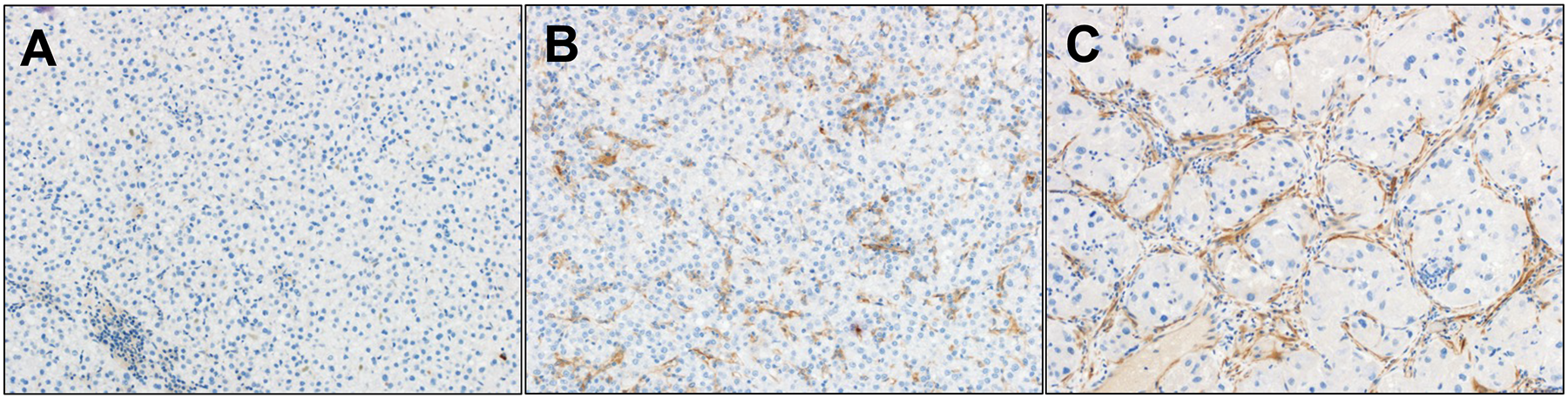

Supplement: Supplemental Figure S1 — FAP expression in metastatic hepatocellular carcinoma (mHCC) (A-C). Negative staining (A), weak positive staining (B), strong positive staining (C). Original magnification (20x). [file Image1.tif]

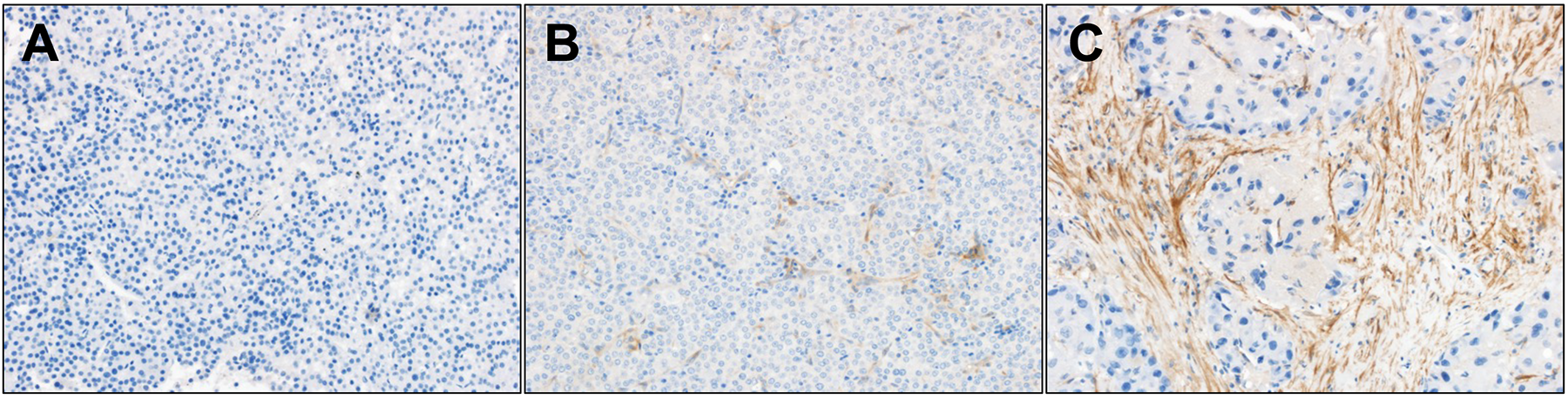

Supplement: Supplemental Figure S2 — FAP expression in hepatocellular carcinoma (HCC) (A-C). Negative staining (A), weak positive staining (B), strong positive staining (C). Original magnification (20x). [file Image2.tif]

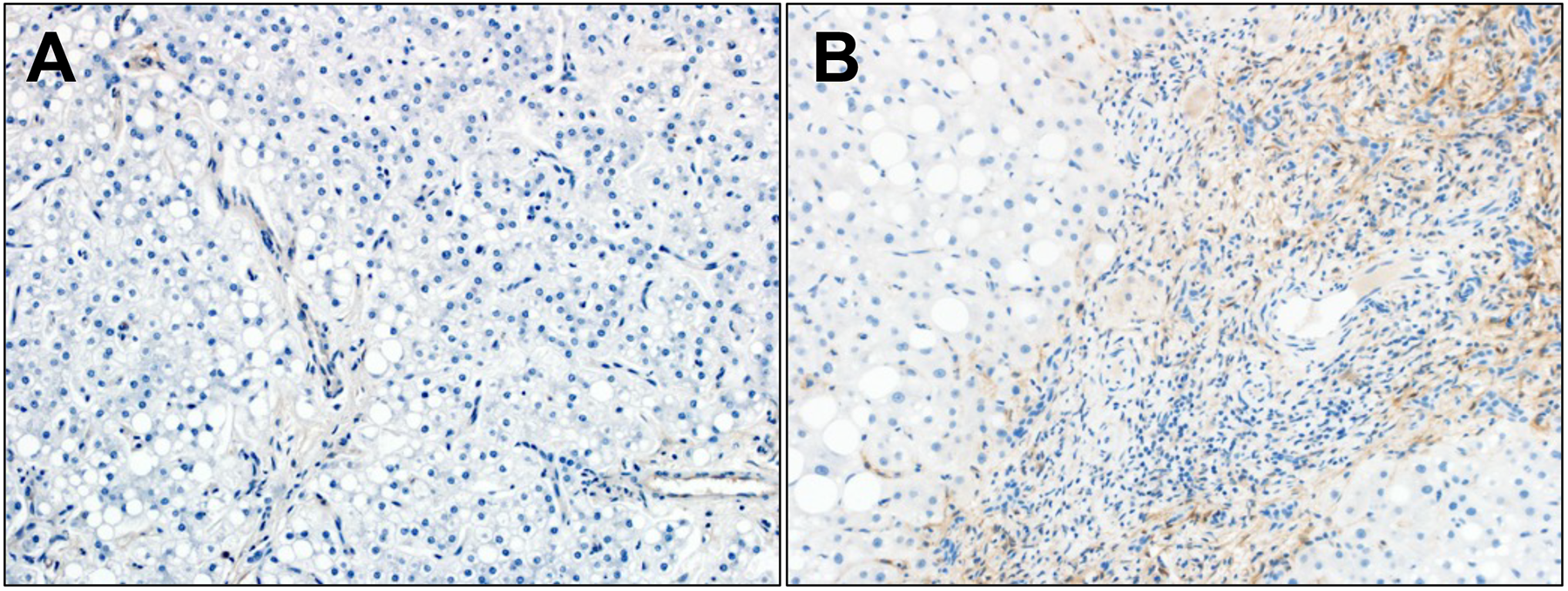

Supplement: Supplemental Figure S3 — FAP expression in fibronodular hyperplasia (A, B). Negative staining (A), moderate positive staining (B). Original magnification (20x). [file Image3.tif]

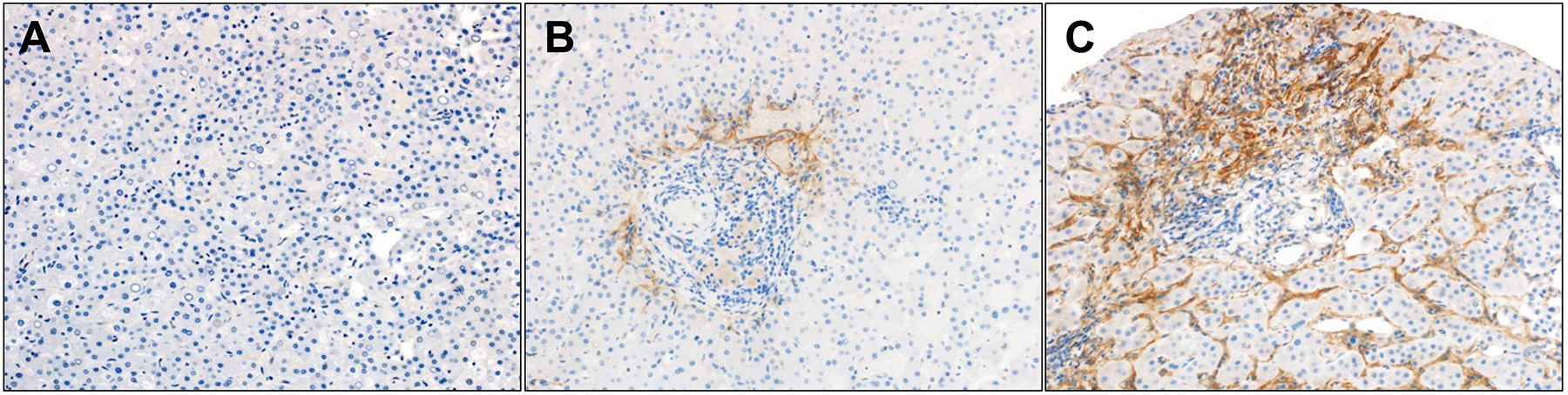

Supplement: Supplemental Figure S4 — FAP expression in hepatic adenoma (HCA) (A-C). Negative staining (A), weak positive staining (B), strong positive staining (C). Original magnification (20x). [file Image4.tif]
